# Editorial: Editors' showcase: dementia care

**DOI:** 10.3389/frdem.2024.1411549

**Published:** 2024-05-13

**Authors:** Wendy Moyle

**Affiliations:** Menzies Health Institute Queensland, Griffith University, Brisbane, QLD, Australia

**Keywords:** dementia, care, person-centered, caregivers, burden

Dementia is a syndrome that describes the loss of memory, language, problem-solving and thinking abilities that interfere with daily living activities (World Health Organisation, 2023). Although the concept of dementia has been around since early 1906, there has been a shift in more recent years from viewing dementia as being pathologically related to aging to a distinct condition. However, although the research focus has remained on finding a cure, there is no current cure. As a result of an emphasis on cure, less emphasis has been placed on the impact and evidence-based basis of caregiving (Ballenger, 2017), which may have reduced the opportunities for people with dementia and their caregivers to have access to quality dementia care.

Dementia care refers to care of all dementia conditions throughout the stages of dementia. Dementia care provides targeted assistance and support to a person with dementia and their caregiver. A caregiver, also known as a carer, is an individual who provides care for a person living with dementia. Such a person is usually a friend or a family member. Approximately 11 million Americans (Alzheimer's Association, 2022) and at least 140,900 informal Australian primary carers care for people with dementia (Australian Institute of Health Welfare, 2024). Providing care can be overwhelming for caregivers at times. We must use evidence-based interventions to provide care to reduce caregiver burden. Although person-centered care is promoted to provide quality dementia care (Fazio et al., 2018), other evidence-based interventions must be considered and investigated to achieve positive outcomes. The six articles in this Research Topic address dementia care interventions in various ways, provide evidence, and describe novel approaches to dementia care.

Bushell et al. report on the effectiveness of educational interventions in the community that aim to improve informal carers' knowledge of dementia anatomy, physiology, progression, and their impact on behavior. They suggest that when caregivers do not understand the behavioral changes regarding dementia, this can result in a higher caregiver burden. The authors' systematic review demonstrates limited education interventions that incorporate information on the anatomy and physiology of the brain. As a result, the authors conclude that more rigorous studies are required to investigate the effect of education interventions on caregivers' dementia knowledge and their role in dementia care.

Sadak and Borson used a cross-sectional mixed-methods descriptive study to illustrate an inductive Six Domain Framework and simple assessment tools used in a sample of dementia dyads. Care partners and their care recipients (*n* = 88) from three university-affiliated hospitals participated. Care recipients' diagnoses, medications, and care records were extracted from the hospital's electronic medical records. Over one-third of care partners reported stress, depression or anxiety, and one-fifth were positive for poor health and social needs. Furthermore, care recipients' (68%) records reported chronic conditions. The Framework was considered suitable for research concerning pathways for dementia care.

The importance of technologies in dementia care is presented in two manuscripts by Wong and Hung and Salai et al. Wong and Hung demonstrate an example of the cultural adaptation of a television video regarding drinking water for older Chinese adults with dementia. They used the cultural adaptation process model to guide the adaptation process. Salai et al. explored a prototype voice assistant called IntraVox that uses a personalized human voice to send prompts and reminders. Their qualitative study reported the benefits of using voice assistants to assist with dementia care. The IntraVox pilot study demonstrated that the device allowed the person with dementia to continue to be cared for at home. These manuscripts are potent reminders of the importance of technology in assisting caregivers in providing care for people with dementia in the community.

Sussman and Tétraulta's qualitative interpretive study collected data regarding advanced care planning (ACP) for people with dementia from 17 community-based staff in three focus groups. Four key barriers were identified: (1) the stigma associated with dementia, (2) lack of knowledge about end-of-life concerns, (3) uncertainties about managing complex family dynamics, and (4) worries that opening conversations about future care may lead to expression of a person's wishes that could not be actualized. The importance of understanding ACP for people with dementia is outlined through these findings.

The final manuscript reported a narrative review that explored the conscious experience and communication modalities used by people with dementia and considered the heightened emotional states experienced as compensatory mechanisms (Warren). Communication difficulties are challenging for people with dementia and their caregivers (Moyle, 2023). An understanding of emotional processing is thought to provide clinical insight into the subjective experience of dementia and, as a result, improve empathy and communication. Both concepts are important in the provision of person-centered care.

In summary, the manuscripts included in this Research Topic introduce several dementia care interventions and theoretical concepts that highlight the importance of understanding the human condition regarding dementia to provide quality dementia care. The manuscripts demonstrate the diversity of the topic of dementia care and the opportunities that can improve the care of the person with dementia and reduce the caregiving burden.

## Author contributions

WM: Writing – original draft, Writing – review & editing.
